# Selected β^2^-, β^3^- and β^2,3^-Amino Acid Heterocyclic Derivatives and Their Biological Perspective

**DOI:** 10.3390/molecules26020438

**Published:** 2021-01-15

**Authors:** Urszula Bąchor, Marcin Mączyński

**Affiliations:** Department of Organic Chemistry, Faculty of Pharmacy, Wroclaw Medical University, 50-556 Wroclaw, Poland; marcin.maczynski@umed.wroc.pl

**Keywords:** β-amino acid derivatives, heterocyclic compounds, antiviral, anti-inflammatory, antibacterial, ALK inhibitors, agonists and antagonists of receptors

## Abstract

Heterocyclic moieties, especially five and six-membered rings containing nitrogen, oxygen or sulfur atoms, are broadly distributed in nature. Among them, synthetic and natural alike are pharmacologically active compounds and have always been at the forefront of attention due to their pharmacological properties. Heterocycles can be divided into different groups based on the presence of characteristic structural motifs. The presence of β-amino acid and heterocyclic core in one compound is very interesting; additionally, it very often plays a vital role in their biological activity. Usually, such compounds are not considered to be chemicals containing a β-amino acid motif; however, considering them as this class of compounds may open new routes of their preparation and application as new drug precursors or even drugs. The possibility of their application as nonproteinogenic amino acid residues in peptide or peptide derivatives synthesis to prepare a new class of compounds is also promising. This review highlights the actual state of knowledge about β-amino acid moiety-containing heterocycles presenting antiviral, anti-inflammatory, antibacterial compounds, anaplastic lymphoma kinase (ALK) inhibitors, as well as agonist and antagonists of the receptors.

## 1. Introduction

Heterocycles are cyclic compounds having atoms of at least two different elements as ring members. Most of the naturally occurring heterocycles, e.g., antibiotics belonging to the penicillin group, alkaloids such as vinblastine, morphine, reserpine, etc., have a heterocyclic moiety [1]. Till now, a wide range of synthetic heterocycle preparation methods has been developed using both solution and solid-phase synthesis. This class of organic compounds presents a wide range of applications. Predominantly, they are used as pharmaceuticals, agrochemicals and veterinary products [2]. They were also successfully applied as sanitizers, developers, antioxidants, corrosion inhibitors, copolymers and dyestuff. Heterocycles are promising agents for drug design as they can be easily synthesized in laboratories by using routine synthetic methods. The location of common heteroatoms, including nitrogen, oxygen and sulfur, is different; however, most of the heterocycles can be divided into groups characterized by the specific structural motif [3]. One of them is a β-amino acid moiety, which often plays a crucial role in their biological activity.

**Figure 1:**
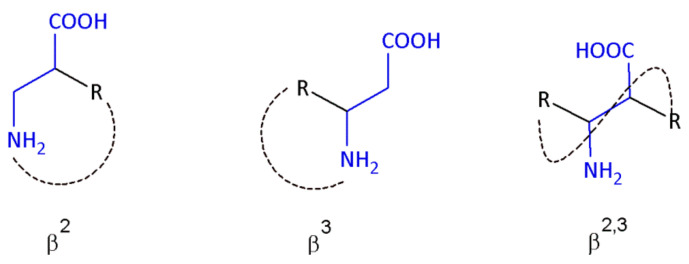
Schematic presentation of different β-amino acids.

β-amino acids are nonproteinogenic amino acids, in which the amino group is present on the carbon atom at the β position to the carboxy group, and depending on the position of the side chains on the 3-aminoalkanoic acid skeleton, can be divided into β^3^-, β^2^-, and β^2,3^-amino acids (Figure 1) [4].

**Figure 2:**
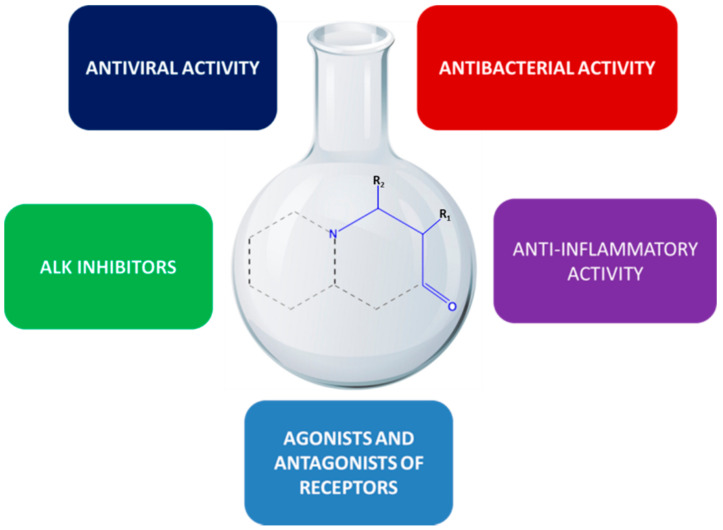
Schematic presentation of the biological activity of heterocycles with β-amino acid moiety.

The signification of β-amino acids has led to an increase in the number of synthetic methods for the preparation of that class of compounds [5,6,7]. Special attention has been paid to β-amino acid-based drug research in the field of medicinal chemistry (Figure 2). Over the past decade, β-amino acid moiety has been successfully used in the design of bioactive ligands and novel biomaterials [8]. Usually, they are incorporated into β-peptides or other β-amino acid-containing oligomers, which may serve as promising tools in medicinal chemistry due to their enhanced biological stability, especially in the case of common proteolytic enzymes, arising from the lack of any interaction between them and in vivo enzymes [9]. A large number of β-amino acids, e.g., β-amino acid with a pyrrolidine core, has been used as building blocks in the development of novel peptides exhibiting antimicrobial activities or being selective inhibitors of the TNF-α converting enzyme [10]. It is known that combinations of two or more biologically active compounds may provide to obtain new molecules with potential strengthened biological activities. Taking into consideration, Venepally et al. have designed and obtained heterocyclic-fatty acid hybrid molecules that exhibited anticancer and antimicrobial activities [11]. The biological analysis of fatty acid 3,4-dihydropyrimidinones (DHPM-fatty acids) obtained by Treptow et al. demonstrated that these types of compounds present antiproliferative activities on glioma cell lines (C6 rat and *U-*138-MG human) [12]. β-Amino acids are now of scientific interest due to their application in the synthesis of peptide mimetics and certain new biologically active substances. They are a constituent of some toxins and a special class of pore-forming lipopeptides, which exhibit antibacterial and antifungal activity. Peptidomimetics containing β-amino acids represent one of the strategies in developing therapeutic candidates with increased oral bioavailability and metabolic degradation resistance. Consequently, β-amino acids are potential inducers of secondary structures, and even single β-amino acid residues stabilize discrete conformations in cyclic peptides [13]. When incorporated into the peptide sequence to improve proteolytic stability, highly functionalized cyclic β-amino acid moieties may have a significant impact on drug design [14].

Heterocyclic β-amino acids can be divided into two types, including 1-aza-polycyclo-2-acetic acid and 1-azapolycyclo-3-carboxylic acid derivatives [13]. Although such compounds and their derivatives are not very common in nature, some examples such as homoproline, capreomycidine, tubulysine or streptolidine are known to exist. The most well-known heterocyclic β-amino acids include the 5-membered pyrrolidine and 6-membered piperidine derivatives. They are present in some alkaloids, peptides and other biologically active compounds.

Heterocycles with a β-amino acid moiety can be found in compounds exhibiting antiviral properties. Some known structures act against the influenza virus [15]. Neuraminidase, a glycoprotein expressed on the influenza virus’ surface, is crucial for virus replication and infectivity by breaking the linkage between the progeny virus on the surface of the host cells’ sialo-receptors. This is a key factor in discovering new anti-influenza drugs that can inhibit neuraminidase, like tri- and tetrasubstituted pyrrolidine derivatives [16].

Our research team has also recently presented a group of heterocyclic compounds containing a β-amino acid motif presenting different biological activities, and their potential therapeutic usefulness has been evaluated as well [17]. The MZO-2 compound, an isoxazole β-amino acid derivative, suppressed LPS (lipopolysaccharide)-induced production of TNF α (tumor necrosis factor α) [18].

In the context of antimicrobial resistance and the widespread use of antibiotics, it is still a current issue. In the case of the compounds described, attention was paid to antibacterial molecules. Ofloxacin, morfloxacin, gatifloxacin, or gemifloxacin are some examples of quinolone antibiotics that are used for treating many bacterial infections [19].

β-amino acid-containing heterocycles are present in several drugs that act by being either agonists or antagonists of the receptors that respond to neurotransmitters, which are chemical messengers. It is known that the selective agonism of A_3_ adenosine receptors is associated with anticancer [20], cardioprotective [21,22] and cerebroprotective activity [23,24] while the selective antagonists [25] for A_3_ adenosine receptors are of potential clinical use for combating inflammation and asthma. Due to this, A_3_ adenosine receptors are still promising targets for the development of clinically useful molecules.

The biological potential of heterocycles possessing β-amino acid motifs also has found application in searching for new anaplastic lymphoma kinase (ALK) inhibitors for the treatment of non–small-cell lung cancer (NSCLC) [26].

Although there are known heterocycles as well as β-amino acids presenting biological activity since today, there is no work that focuses on the types of compounds that contain both moieties.

Since heterocycles are not usually considered β-amino acid moiety-containing compounds, there is a strong need to present the overview of the actual state of knowledge of this class of biologically active compounds, which is the main topic of this manuscript.

## 2. Biological Activity

### 2.1. Antiviral Activity

Influenza is one of the most important health problems. There is still a need to develop new anti-influenza drugs to ensure the appropriate bioavailability of drugs for treating a broad spectrum of influenza viruses, including seasonal flu and possible pandemic infections. All influenza viruses bear two surface glycoproteins, hemagglutinin and neuraminidase, which are the antigens that define the particular influenza strain. The hemagglutinin is a sialic acid receptor–binding molecule and mediates the entry of the virus into the target cell. Neuraminidase (sialidase) enzymes are glycoside hydrolases that cleave α-ketosidic linkage between the sialic acid (*N*-acetylneuraminic) and an adjacent sugar residue to which the newly formed particles are attached. This cleavage enables the virus to be released from the host’s cells. When inhibiting neuraminidase, infection would be limited to one round of replication, rarely enough to cause disease. Neuraminidase may also facilitate viral invasion of the upper airways, possibly by cleaving the sialic acid moieties on the mucin that bathes the airway epithelial cells. The first neuraminidase inhibitors to be designed were DANA (2-deoxy-2,3-didehydro-*N*-acetylneuraminic acid) and FANA (2-deoxy-2,3-dehydro-*N*-trifluoroacetylneuraminic acid) [27]. They served as the lead structures in the development of the new neuraminidase inhibitors known as zanamivir and oseltamivir [28]. However, a mutation of the influenza virus resulted in its resistance to these drugs, which prompted the development of neuraminidase inhibitors containing a β-amino acid moiety for the influenza virus. Wang et al. [29] started from the initial **A-87380** (Table 1, Compound **1**, (3*S*,4*S*)-4-amino-1-(*tert*-butoxycarbonyl)-pyrrolidine-3-carboxylic acid), belonging to the β^2,3^-amino acid heterocyclic derivatives as other compounds presented in Table 1, which acted as a modestly active neuraminidase inhibitor with IC_50_ = 50 μM. In the chemical structures of biologically active compounds presented below, the β-amino acid moiety is highlighted in blue.

The authors concluded that the synthesized compound **A-87380** might serve as a valid lead and set out to optimize the structure to discover clinically effective oral neuraminidase inhibitors. The tri- and tetrasubstituted pyrrolidine moiety-containing derivatives were obtained using combinatorial chemistry techniques, particularly high-throughput parallel synthesis, leading to the discovery of **A-192558** (Table 1, Compound **2**). Both of the presented compounds contained a β-amino acid moiety.

Some substrates mimic the inhibitors of influenza virus neuraminidase, for example, the thioglycoside, 3-deoxy-3-fluoro derivative and phosphonate of sialic acid (Neu5Ac), whereas the 2-deoxy-2,3-didehydro-*N*-acetylneuraminic acid (Neu5Ac2en, DANA) was identified as the first effective neuraminidase inhibitor (K_i_ = 4 × 10^−6^ M) which mimics the transition state in the enzymatic cleavage of the sialo-moiety.

Other examples of heterocyclic β-amino acid-derivative agents to be used against viruses were designed by using a chiral cinchona alkaloid-squaramide catalyst. Bai et al. [30] obtained a series of both enantiomers of novel aminopyrimidine derivatives in an enantioselective three-component one-pot Mannich reaction. Additionally, chiral compounds—Compound **3** and Compound **4** (Table 1)—exhibited higher antiviral activities against tobacco mosaic virus (TMV) in vivo than the commercial agent ningnanmycin (concentration of 500 μg/mL, with a curative activity of 56.8% and 55.2%, a protection activity of 69.1% and 67.1%, and an inactivation activity of 91.5% and 94.3%, respectively). The antiviral mechanisms and enhanced antiviral activities of these chiral derivatives are interesting subjects for future research.

To identify the 1*H*-pyrazolo-[3,4-*b*]-pyridine system with the incorporated β-amino acid moiety derivative as promising compounds for the development of new antiviral agents, Bernardino and colleagues obtained new.

4-(phenylamino)/4-[(methylpyridin-2-yl)-amino]-1-phenyl-1*H*-pyrazolo-[3,4-*b*]-pyridine-4-carboxylic acids derivatives [31]. Ethyl-4-chloro-1-phenyl-1*H*-pyrazolo-[3,4-*b*]-pyridine-5-carboxylate reacted with the appropriate substituted anilines to give the new ethyl 4-(phenylamino)-1-phenyl-1*H*-pyrazolo[3,4-*b*]-pyridine-5-carboxylate (Table 1, Compound **5**) required.

Hydrolysis of the esters afforded the corresponding carboxylic acids (Table 1, Compound **6**). The anti-herpetic effect of all compounds investigated was evaluated in a primary screening using the 50% end-point titration method. In the group of esters, Compound **5** (Table 1) exhibited the highest anti-HSV-1 activity, with the esters generally being more effective inhibitors than the corresponding acid. This observation led authors to conclude that the introduction of the ester group at C-5 in the 1*H*-pyrazolo-[3,4-*b*]-pyridine system increases the activity against the HSV-1 virus. Authors infected Vero cells (lineage of cells used in cell cultures) with HSV-1, Mayaro virus, and VSV (vesicular stomatitis virus) to calculate the selective or therapeutic index of each compound for antiviral activity. Other derivatives (Table 1, Compound **6*:*** 1-phenyl-4-(phenylamino)-1*H*-pyrazolo-[3,4-*b*]-pyridine-5-carboxylic acid derivatives) exhibited the best EC_50_ values against the VSV and Mayaro virus replication cycles while in the case of Compound **5** (Table 1) it was against HSV-1.

Pyrazole analogs are useful building blocks in designing pharmaceuticals. In comparison with the other tested compounds, Compound **7** manifested the highest anti-HAV (hepatitis-A virus) activity at a concentration of 20 μg/105 cells [32].

Salem et al. [33] used a facile one-pot four-component reaction to obtain 2-oxo-1,2-dihydropyridine-3-carbonitryle as a substrate to design new heterocyclic systems and evaluated them as antiviral agents. Some of the synthesized heterocycles demonstrated promising antiviral activities toward the rotavirus Wa and adenovirus type 7 strains. Compound **8** showed a 60% and 53.3% reduction in viral titer with rotavirus Wa strain and adenovirus type 7, respectively.

Sriram et al., synthesized a series of nevirapine derivatives with a broad-spectrum of chemotherapeutic properties for the effective treatment of HIV/AIDS [34]. Compound **9**, containing an unsaturated β-amino acid moiety, showed 100% inhibition against *Mycobacterium tuberculosis* and also presented strong antibacterial activity against 24 pathogenic bacteria with MIC less than 1 µg/mL. The antiviral test was carried out using Vero cells, HSV-1, and aphidicolin (0.005 µg/mL) as a positive control.

Attention was also paid to antiviral β-amino acid ester phosphodiamide derivatives, the formula of which is presented below (Compound **10**). Their pharmaceutically acceptable salts are useful for the inhibition of ΗΓν reverse transcriptase. They may also be useful for the prevention or treatment of ΗΓν infection, as well as prevention, treatment and delaying the onset or progression of AIDS [35].

The retrovirus-designated human immunodeficiency virus (HIV) (strains HIV-1 and HIV-2) have been etiologically combined with this immunosuppressive disease and acquired immunodeficiency syndrome (AIDS). Initially, HIV seropositive patients are asymptomatic but typically develop AIDS-related complex followed by AIDS. The individuals affected exhibit high immunosuppression, which makes them extremely susceptible to debilitating and ultimately fatal opportunistic infections. The replication of HIV by a host cell follows the integration of the viral genome into the host cell’s DNA. Inhibition of reverse transcriptase (RT, enzyme that catalyzes the transcription of retrovirus RNA into DNA) inhibits HIV replication in infected cells. There are two classes of reverse transcriptase inhibitors: one is non-nucleoside active site competitive RT inhibitors, such as Nevirapine, and the other is active site RT inhibitors, which comprise nucleoside reverse transcriptase inhibitors (NsRTIs) and nucleotide reverse transcriptase inhibitors (NtRTIs), collectively referred to as NRTIs (tenofovir disoproxil fumarate) [36].

In other research to identify new HIV-1 inhibitors, Corona et al., obtained some new 4-amino-5-benzoyl-*N*-phenyl-2-(substituted-amino)-1*H*-pyrrole-3-carbothioamide derivatives (Table 2, Compound **11**) and tested them against RNase H activity [37]. In the group of the synthesized compounds, some exhibited IC_50_ values in the low micromolar range. The authors concluded that the 4-methylbenzoyl moiety in the 2-amino group led to compounds that showed IC_50_ values ranging from 6 to 8 μM (Compound **11**, **A1**–**A6**), except for 4-chlorophenylamino and 3,4-dichlorophenylamino derivatives. Since only the 4-phenylamino derivative **A8** among the Compound **11**, **A7**–**A13** retained some RNase H inhibition properties (IC_50_ of 19 μM), the replacement of the 4-methylbenzoyl group with a 4-methoxybenzoyl group caused a loss of activity. However, the presence of 4-chlorophenylamino group in the case of the 4-chlorobenzoylpyrrole series (**A14**–**A17**) did not affect inhibition of RNase H. Finally, the replacement of the aroyl moiety with a small alkylamino group (**A18**) or a pyridylamino moiety (**A20**) was detrimental for the anti-RNase H activity in the pyrrole derivatives bearing an unsubstituted benzoyl moiety, while its replacement with a phenyl acetyl moiety (**A19**) preserved the activity.

### 2.2. Anti-Inflammatory

Nonsteroidal anti-inflammatory drugs (NSAIDs) are used all over the world for their analgesic, anti-inflammatory, and antipyretic effects [38]. These drugs have a therapeutic effect as they act as inhibitors of cyclooxygenase (COX), the enzyme that produces prostaglandins. They cause—to a greater or lesser degree—the same side effects, including gastric and renal toxicity. Recent studies have shown that there are at least two types of COX isoenzymes: COX-2 and COX-2. COX-1 is constitutive and produces prostaglandins that protect the stomach and kidneys from damage. COX-2′s mechanism of action is the production of prostaglandins (that are responsible for pain) when induced by inflammatory stimuli, such as cytokines. Therefore, selective COX-2 inhibitors should be anti-inflammatory without side effects on the kidneys and stomach (although selective COX-2 inhibitors may cause other side effects). For example, COX-2 is thought to be involved in ovulation and labor. Additionally, the well-known protective effect of aspirin on colon cancer may be through the way it affects COX-2, which is expressed in this disease. It is also known that NSAIDs delay the progress of Alzheimer’s disease. Thus, selective COX-2 inhibitors are thought to be promising agents in many diseases [39].

Zomerpirac (Table 3, Compound **12**), a prostaglandin synthetase inhibitor, is the sodium salt of 5-(4-chlorobenzoyl)-1,4-dimethyl-1*H*-pyrrole-2-acetate dehydrate, containing β-amino acid moiety. It is a pyrrole-acetic acid that is structurally related to tolmetin (Table 3, Compound **13**). Studies demonstrated zomepirac to be more effective than aspirin or codeine alone and to be as effective as analgesic combinations containing codeine or other opioids [40]. Tolmetin converts arachidonate into prostaglandin H2 (PGH2), a committed step in prostanoid synthesis. In patients with rheumatoid arthritis or osteoarthritis, tolmetin inhibited disease activity similarly to aspirin but caused more gastrointestinal side effects [41]. Those two compounds are examples of β^3^-amino acid heterocyclic derivatives. Other compounds presented in Table 3 belong to β^2,3^-amino acid heterocyclic derivatives.

Tolmetin restrains prostaglandin synthetase in vitro and reduces prostaglandin E plasma levels, possibly causing the anti-inflammatory response. The side effects that tolmetin may cause include an increased risk of heart or circulatory conditions such as heart attacks and strokes.

The activity of another compound containing a β-amino acid moiety, ketorolac (the brand name Toradol**,** Table 3, Compound **14**), is related to blocking cyclooxygenase 1 and 2 (COX-1 and COX-2), thereby decreasing the production of prostaglandins. Contraindications to ketorolac are the risk of gastrointestinal bleeding, risk of renal failure, compromised hemostasis, hypersensitivity to aspirin or other NSAIDs, labor, delivery, and nursing [42].

Izoxazole derivatives exhibiting anti-inflammatory properties are a great group in this class of potential therapeutics [17,43]. Ryng and colleagues focused on the synthesis of 5-amino-3-methyl-4-isoxazolecarboxylic acid as a β-amino acid derivative. In an earlier study, the authors demonstrated strong anti-inflammatory and antibacterial activity of *p*-etoxyphenylamid (Table 3, Compound **15**) and *p*-chlorophenylamid of 5-benzoylamino-3-methyl-4-isoxazolecarboxylic acid (Table 3, Compound **16**). The most potent activity of these compounds was attributed to the benzoyl group in position 5 of the isoxazole ring [44].

A set of new isoxazole derivatives with expected immunosuppressive activities was derived [29]. Following in vitro screening in the human cell models, the activity of a most potent MZO-2 Compound **17** (Table 3) (ethyl *N*-(4-{[(2,4-dimethoxyphenyl)methyl]-carbamoyl}-3-methyl-1,2-oxazol-5-yl)ethanimidate) in mouse in vivo models was evaluated. In vitro tests included evaluation of peripheral blood mononuclear cell (PBMC) viability, phytohemagglutinin (PHA)-induced PBMC proliferation and lipopolysaccharide (LPS)-induced tumor necrosis factor α (TNF α) production in whole blood cell cultures. MZO-2 suppressed LPS-induced production of TNF α. The compound, administered intraperitoneally, inhibited carrageenan-induced footpad edema and contact sensitivity to oxazolone in mice after ointment application, with a potency that was comparable to tacrolimus (Protopic^®^). The compound also inhibited the expression of caspases 3, 8, and 9 in Jurkat cells [45].

### 2.3. Antibacterial

Due to the increasing multidrug resistance of microbial pathogens, the amount of bacterial and fungal infections is currently an important problem [46]. The widespread use of antibiotics has contributed to growing infection rates, as fungal infections occur after antibiotic therapy due to the elimination of beneficial bacteria that normally suppress fungi. There is still a need to develop new effective antifungal and antibacterial agents.

**Figure 3:**
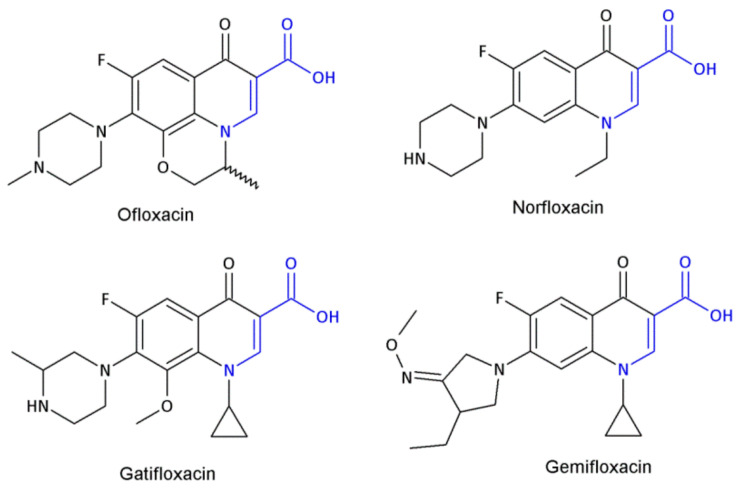
The structures of ofloxacin, norfloxacin, gatifloxacin and gemifloxacin, unsaturated β-amino acid heterocycles exhibiting antibacterial activities.

Ofloxacin, norfloxacin, gatifloxacin, and gemifloxacin (Figure 3) are antibiotics containing a β-amino acid, which are useful for treating many bacterial infections and belong to a class of drugs known as quinolone antibiotics [47,48,49,50]. *Streptococcus pneumoniae* is the main cause of bacterial respiratory tract infections in humans. It causes bronchitis to turn to life-threatening pneumonia, bacteremia and meningitis. Ciprofloxacin and other marketed quinolones exhibit only modest activity against it, limiting their use in the case of patients with serious respiratory tract infections. Ofloxacin is effective against Gram-positive and Gram-negative microorganisms, as well as *Chlamydia trachomatis*. The minimum inhibition concentration (MIC) of ofloxacin, at which 90% of isolates are inhibited for 55 isolates of pneumococci, was 4 μg/mL [47]. Norfloxacin also has a wide spectrum of action; this drug acts against Gram-positive bacteria, i.e., methicillin-susceptible *Staphylococcus epidermidis*, *Staphylococcus saprophyticus*; Gram-negative bacteria: *Enterobacteriaceae*, *H. influenzae,* other *Hemophilus* spp., *N. gonorrhoeae*, *N. meningitides*, *M. catarrhalis*, *P. aeruginosa*. Norfloxacin is used to treat different bacterial infections of the prostate and urinary tract (bladder and kidneys), as well as in eye drops. The new fluoroquinolone derivatives, such as Gatifloxacin and Gemifloxacin, have much to offer in terms of bacterial eradication, including activity against such resistant respiratory pathogens as penicillin-resistant, macrolide-resistant, and multidrug-resistant *S. pneumoniae*. Gatifloxacin was found to produce a clinical cure rate of 91% versus 88% for levofloxacin and cefuroxime axetil in the treatment of acute exacerbations of chronic bronchitis (ECB). The use of Gemifloxacin in the treatment of ECB was studied by Ball et al. [51] and Wilson et al. [52]. Gemifloxacin displayed similar clinical success rates to Trovafloxacin (91% vs. 87.6%) and bacterial success rates of 87% [49].

*Chlamydia pneumoniae* (*Chlamydophila pneumoniae*) is an airborne respiratory tract pathogen that typically causes prolonged, dry cough, pharyngitis and, hoarseness, often accompanied by sinusitis (approximately 70% of acute *C. pneumoniae* respiratory tract infections). Additionally, it is estimated that 5–10% of community-acquired pneumonia cases are caused by *C. pneumoniae*, and elderly patients often suffer from a severe illness upon encountering it [53].

Moxifloxacin (Table 4, Compound **18**) and ciprofloxacin (Table 4, Compound **19**), unsaturated β-amino acid heterocycles, are highly active fluoroquinolones containing a β-amino acid moiety, which are used in the treatment of community-acquired pneumonia caused by *Chlamydia pneumoniae* and chronic prostatitis caused by *Chlamydia trachomatis*, respectively [54,55].

Oxetin (Table 4, Compound **20**), β^2,3^-amino acid heterocyclic derivative*,* is the first amino acid–antibiotic with an oxetane ring containing a β-amino acid moiety [56]. The compound inhibits *Bacillus subtilis,* and *Pyricularia oryzae* exhibits a herbicidal activity and inhibits glutamine synthetase from spinach leaves.

The novel β-d-glucosaminopyranosyl template containing a β^2,3^-amino acid moiety (SAA-sugar amino acid moiety, Table 4, Compound **21**) was prepared [57] using the synthetic strategy developed by Suhara and colleagues [58].

The effectiveness of peptide Compound **21** (Table 4) as an antibacterial agent was assessed by employing a standard minimal inhibitory concentration (MIC) assay against four Gram-positive and two Gram-negative bacterial strains. This compound showed a complete loss of activity against all bacterial strains in this assay. Moreover, the peptide lost all toxicity toward human erythrocytes. Since these results correlate with the abovementioned antimicrobial activity and the same trend for antimicrobial activity and hemolytic activity was observed, it can be concluded that the therapeutic value of the peptides presented in these studies is limited. The authors presented a highly efficient gramicidin S synthesis strategy for the incorporation of nonproteinogenic sugar amino acids, which can be developed in the future in examining new active compounds.

In a search for new biologically active compounds to combat bacteria, Mickevičius et al., synthesized a series of new *N*,*N*-disubstituted β-amino acids and their derivatives with thiazole, aromatic, and heterocyclic substituents [59].

The antibacterial activity of the proposed compounds was determined in the case of their use against Gram-positive spore-forming rods of *Bacillus cereus* (ATCC 11,778), Gram-positive cocci of *Staphylococcus* (ATCC 9144), Gram-negative rods of *E. coli* (ATCC 8739) and *Pseudomonas aeruginosa* (NCTC 6750) using the broth and spread-plate methods. The antibacterial screening data showed a remarkable inhibitory activity of some β-amino acid derivatives of the 3-{[(5*Z*)-5-Substituted 4-oxo-4,5-dihydro-1,3-thiazol-2-yl](phenyl)amino}propanoic acid (Table 4, Compound **22** are some examples of them) and Compound **23** (Table 4) against the tested strains of *S. aureus*, *B. cereus*, *E. coli* and *P. aeruginosa* (MIC = 250 µg/mL).

Among all of the obtained β^3^-amino acid heterocyclic derivatives, the highest antibacterial activity was exhibited by compounds containing bromothiophene, pyrrole and furan substituents (oxytetracycline as a reference drug), whereas a derivative with a tiophene moiety did not show antibacterial activity against the tested microorganisms. Mickevičienė and colleagues converted 3-[(2-hydroxyphenyl)-amino]-butanoic and 3-[(2-hydroxy-5-methyl-(chloro)-phenyl)-amino]-butanoic acids into a series of derivatives containing hydrazide, pyrrole and chloroquinoxaline moieties [60]. The compounds obtained exhibited good antimicrobial activity against *Staphylococcus aureus* and *Mycobacterium luteum*, whereas some compounds showed significant antifungal activity against *Candida tenuis* and *Aspergillus Niger*. Vancomycin was used as a control in the tests of antibacterial activity of the compounds synthesized, and nystatin was used in the antifungal activity tests. At lower concentrations, Compounds **24** (Table 4), Compound **25** and Compound **26** (Table 4) are active against the *S. aureus* and *M. luteum* Gram-positive bacteria. Compounds **24** showed inhibition activity against the *S. aureus* bacterial strain at concentrations of 62.5 µg/mL, 31.2 µg/mL and 31.2 µg/mL, respectively. Compound **25** was active at 31.2 µg/mL, and Compound **26** at 62.5 µg/mL. Only Compound **24c** and Compound **27** (Table 4) showed inhibition against *E. coli.* (concentration of 500 µg/mL). Some compounds inhibited the growth of *M. luteum*: Compounds **24a**–**c** at 62.5 µg/mL, Compound **25** at 31.2 µg/mL, Compound **26** at 62.5 µg/mL, Compound **27** at 62.5 µg/mL. Antifungal activity against *C. tenuis* was observed only for three compounds (e.g., Compound **25a**) and some inhibited growth of *A. Niger* microorganisms (e.g., Compound **24**, Compound **26** and Compound **27**).

Structures derived from heterocyclic β-amino acids also can be found in the group of β-lactamase inhibitors. This aspect became very interesting since bacteria developing resistance to β-lactam antibiotics has been established as a major threat to human health in the 21st century [61]. The basic mechanism of resistance to penicillins and cephalosporins is the production of β-lactamase enzymes by bacteria. β-lactamases are a class of the serine-amidase group of enzymes that have been widely studied, with their primary mode of action being the hydrolysis of the β-lactam ring of the antibiotic, leading to a ring-opening and inactivation of the drug [62].

There are two strategies to counter this resistance based on the synthesis of new β-lactam antibiotics that can resist the enzymatic hydrolysis and following deactivation, combined with the obtainment of β-lactamase inhibitors, to be delivered in concert with the currently available antibiotics.

Due to the second strategy, boronic acids that act as transition-state inhibitors have proven to be the most effective reversible inhibitors, presenting a greater intracellular efficacy than phosphonate inhibitors [63]. The boron atom (electrophile) mimics the carbonyl functionality of the β-lactam; the boron forms a tetrahedral geometry with the catalytic serine, imitating the transition state of the enzyme-adduct complex, and hence, blocking access to the active site of the drug molecule β-lactam ring. Boronic acids act as competitive inhibitors and have not been shown to be hydrolyzed by any β-lactamase to date [64,65].

Tondi et al., obtained a set of boronic β^2,3^-amino acid derivatives acting as AmpC β-lactamase inhibitors [66]. The lead Compound **28** (Table 4), containing β-amino acid moiety, presents improved drug-like properties, and with its strong binding to AmpC, it is a promising candidate for further development of this series of compounds with the ability to cross the outer membrane of Gram-negative bacteria. Furthermore, the binding orientation adopted by Compound **28** in AmpC suggests the gain of additional interactions with such surrounding polar residues as Asn289 and Asn343 through the introduction of functional groups at the inhibitor distal phenyl ring.

Shoichet’s group published its findings [67] concerning the field of boronic β^2,3^-amino acid-based β-lactamase inhibitors, and based upon this previous experience, illustrated that the closer the boronic acid resembles the natural substrate, the better its potency. An example of such an inhibitor is Compound **29** (Table 4), which can inhibit some different serine β-lactamases, including *K. pneumoniae* β-lactamases KPC-2 and SHV-1, by interacting with the conserved features of these active sites, which includes forming the bond with catalytic serine via the boron atom, positioning one of the boronic acid oxygens in the oxyanion hole, and utilizing its amide moiety to perform conserved interactions across the width of the active site [68].

G. P. Suresha et al. [69] synthesized a series of novel urea/thiourea derivatives of quinazolinone–lysine conjugates as β^2,3^-amino acid derivatives (Table 4, Compound **30**) and screened them for in vitro antimicrobial activity. The activity analysis results showed that compounds containing urea and thiourea derivatives increased biological activity compared to the standard ones. It is worth noting that the fluoro group attached to the phenyl ring of the conjugates acts as an active moiety in arresting the growth of the microbes so that the nature of the substituent was found to be vital to improving the antimicrobial activity.

Serpins (**ser**ine **p**rotease **in**hibitors) are the greatest family of protease inhibitors that inhibit enzymes by conformational change. They play a crucial role in controlling important proteolytic cascades, including the mammalian coagulation pathways. Serine protease inhibitors are conformationally labile, and many of their disease-linked mutations result in misfolding or pathogenic, inactive polymers [70]. Serine proteases, serine protease inhibitors and protease-activated receptors have been investigated for their effects in coagulation, hemostasis and hemodynamics, inflammation and wound healing. It was observed that serine proteases, their zymogen precursors and endogenous inhibitors, as well as protease-activated receptors substrates, have an impact on synaptic function and behavior. Furthermore, the unnatural activity of these molecules contributes to many neurological disorders such as Alzheimer’s and Parkinson’s disease, and traumatic brain injury [71]. Compound **31** (Table 4) [72] is an interesting example of a β^3^-amino acid moiety-containing boronic acid derivative that acts as a serine protease inhibitor.

### 2.4. ALK Inhibitors

Non-small-cell lung cancer (NSCLC) is the leading cause of cancer-related deaths worldwide. In most NSCLC patients with an advanced or incurable disease, cytotoxic chemotherapy usually results in low response rates and only modest improvements in overall survival. Researchers from Japan identified anaplastic lymphoma kinase (ALK) as another potential target in NSCLC. ALK-positive (anaplastic lymphoma kinase positive, or ALK+) lung cancer occurs in 1 out of 25 non-small-cell lung cancer patients. Generally, patients under 55 years of age who have never smoked are most likely to be diagnosed with ALK+. The ALK mutation is a genetic alteration of lung cell DNA that causes such cells to grow abnormally, and ultimately, behave as cancer cells. ALK inhibitors are anticancer drugs that affect tumors with variations of the anaplastic lymphoma kinase, such as an EML-ALK translocation [26,73].

**Figure 4:**
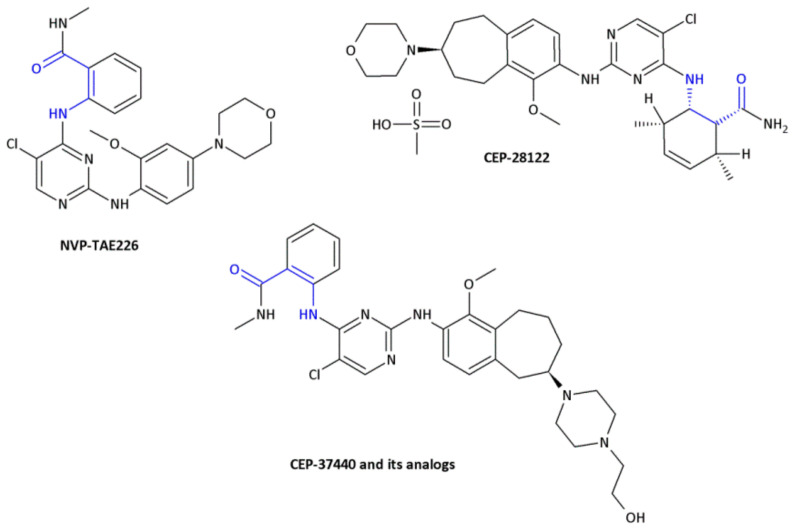
The structures of NVP-TAE226, CEP-28122 and CEP-37440 and its analogs that are ALK inhibitors [69].

Since crizotinib (Xalkori, Pfizer) showed a substantial objective response rate and remarkable progression-free survival, it was the first-in-class tyrosine kinase inhibitor approved by The Food and Drug Administration (FDA). Still, the acquired resistance to crizotinib—particularly observed in the central nervous system, which remains the most common site of relapse—remains a major problem. Searching for new active compounds led to the discovery of numerous next-generation ALK inhibitors, and surprisingly, most of them are 2,4-diarylaminopyrimidine analogs (named DAAPalogues) [74]. Among these compounds, there are also some heterocyclic β^2,3^-amino acid derivatives; CEP-28122 mesylate salt, CEP-37440 (and its analogs) and NVP-TAE226 are some examples of ALK inhibitors (Figure 4, Compound **33**). CEP-28122 is a highly potent and selective orally active ALK inhibitor with IC_50_ = 1.9 ± 0.5 nM in an enzyme-based TRF assay.

**Figure 5:**
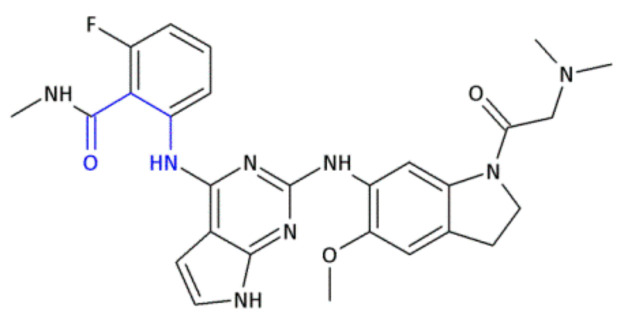
The structure of GSK1838705A [72].

GSK1838705A (Figure 5, Compound **34**) inhibits ALK, with an IC_50_ of 0.5 nmol/L resulting in the complete regression of ALK-dependent tumors in vivo at well-tolerated doses. Based on this, GSK1838705A is a promising antitumor agent for therapeutic use in human cancers [75,76].

### 2.5. Agonists and Antagonists of Receptors

Adenosine contains endogenous purine nucleoside that occurs naturally in body cells and regulates the functioning of the heart. Adenosine opens the potassium channel and prevents the opening of a free calcium channel, serving as an indirect calcium antagonist. As a result, conduction in the atrioventricular node of the heart is slowed down, and the function of the sinus node is inhibited. The drug regulates the work of the heart and prevents atrial fibrillation [77]. Patients undergoing cardiovascular surgery have an increased risk for a myocardial-damaging ischemic event during or within a few days after the procedure [78]. Selective adenosine A_3_ agonists are useful for the prevention of perioperative myocardial ischemic injury. Additionally, noncardiovascular surgeries involving patients with cardiovascular disease risk factors also carry an increased incidence of mortality and morbidity, which is associated with ischemic injury [79]. It has been shown that A_3_ receptor agonists are cardioprotective without inducing hemodynamic effects in a rabbit model of ischemic injury [80].

In an investigation of a highly selective agonist of the human adenosine A_3_ receptor, DeNinno et al., synthesized different amino nucleosides containing a β^2,3^-amino acid motif. Among all of the obtained compounds, Compound **32** and Compound **33** (Table 5) exhibit moderate levels of selectivity and potency [81], whereas CP608039 (Table 5, Compound **34**) binds to the human A_3_ receptor with a K_i_ of 5.8 nM and possesses over 1000-fold selectivity versus the human A_1_ receptor. The introduction of an amino group at the 3′ position of the nucleoside improved not only the selectivity but also the aqueous solubility of the synthesized derivatives.

Based on the previous investigations, DeNinno et al., discovered a highly potent and selective series of adenosine A_3_ agonists [82]. The obtained compounds, containing a β-amino acid moiety, were highly soluble in water (necessary for the intended parenteral administration route) due to the presence of one or two basic amine functional groups. Among a series of compounds, an analog of CP608039—Compound **35** (Table 5)—was shown to be a potent, full agonist of the A_3_ receptor (EC_50_ = 8.1 nM in the test of inhibition of the isoproterenol-induced increase in cAMP in HEK293 cells expressing the appropriate human receptor).

Jeong and colleagues developed the synthesis of novel 3′-ureidoadenosine derivatives, starting from 1,2:5,6-di-*O*-isopropylidene-d-glucose with the presence of the β^2,3^-amino acid motif (Table 5, Compound **36**) [83]. Although they did not discover new and potent A_3_ adenosine receptor agonists, the authors explained the unfavorable steric and electrostatic interactions likely to occur upon binding of the 3′-ureido derivatives in the agonist binding site of the A_3_ adenosine receptor using molecular modeling, which could provide valuable information about the identification of the binding site of the A_3_ adenosine receptor.

It is known that there are some modifications to the adenosine scaffold that increase A_3_ binding affinity and selectivity among adenosine agonists: a 5′-uronamide moiety (as in Compound **37**, Table 5) and N^6^ -positions (as in Compound **38**, Table 5) [84]. In the 3′-amino series, the most potent compound also exhibited 300-fold selectivity over the A_1_ adenosine receptor.

Van Rompaey et al., investigated the influence on affinity, selectivity and intrinsic activity of combined modifications at the 3′- and 5′-positions of the ribofuranosyl moiety with purine modifications at the 2- and N^6^-positions. Among the obtained synthetic analogs, Compound **37** containing a β^2,3^-amino acid moiety, displayed good selectivity and moderate-to-high affinities to the adenosine A_3_ receptor (K_i_ = 41 nM, K_i_ = 60 nM, respectively). However, the ability to tune the efficacy depending on the substituent introduced at the 3′-position was very interesting. A 3′-amino function (as in Compound **38a** and **38b**) resulted in strong partial agonist activity, whereas modifying the amino group to azide converted these analogs into antagonists.

The opioid receptor-like 1 (ORL-1), recently named NOP, was identified in 1994 as a G-protein-coupled receptor that has a high degree of amino acid sequence homology to classic opioid receptors (δ, μ, and κ) [85,86,87]. As an endogenous ligand of ORL-1, Nociceptin/orphanin (N/OFQ) is a peptide that initiates its function and is involved in pain regulation, cognition, anxiety and the cardiovascular system [88,89,90,91,92,93,94].

To obtain potent and selective non-peptide ORL-1 antagonists, Jona et al., developed an efficient and practical asymmetric synthesis of a new key intermediate for the synthesis of nociceptin antagonists [95].

The new few-step synthesis led to obtaining 1-*tert*-butyl 3-methyl-(3*R*,4*R*)-4-(2-oxo-2,3-dihydro-1*H*-benzimidazol-1-yl)-piperidine-1,3-dicarboxylate, a β^2,3^-amino acid moiety containing the compound (Table 5, Compound **39**), a useful intermediate for the synthesis of nociceptin antagonists.

The idea that *N*-methyl-d-aspartate (NMDA) receptors play a key role in causing seizures led to clinical studies of NMDA receptor-blocking drugs in epilepsy. Finally, it was discovered that another type of an ionotropic glutamate receptor, the AMPA receptor, is the predominant mediator of excitatory neurotransmission in the central nervous system [96]. The AMPA receptor (α-amino-3-hydroxy-5-methyl-4-isoxazolepropionic acid receptor) is one of four types of glutamate receptors that participates in excitatory neurotransmission [97]. The prototype quinoxalindiones, such as NBQX [98] and YM90K [99] and other competitive AMPA receptor antagonist classes, are generally poorly soluble and thus difficult to formulate for clinical use.

3-(2-Carboxypiperazin-4-yl)propyl-1-phosphonic acid (Table 5, Compound **40**) is a potent, selective NMDA glutamate receptor antagonist containing a β^2^-amino acid moiety and exhibiting anticonvulsant properties [100].

Nicotinic acid (niacin, vitamin B3, pyridine-3-carboxylic acid) is one of the most effective therapeutic agents for raising high-density lipoprotein (HDL) levels. Moreover, it also protects from other cardiovascular risk factors by lowering very-low-density lipoprotein (VLDL), low-density lipoprotein (LDL), and lipoprotein(a) plasma concentrations [101,102]. Identifying the high-affinity nicotinic acid receptor GPR109A (HM74A in humans and PUMA-G in mice) was made possible due to the identification of a high-affinity nicotinic acid binding site that was localized to adipose, macrophage, and spleen tissues and appeared to function in a G_i_ protein-coupled manner [103,104]. Despite the therapeutic values of nicotinic acid, there are some side effects like cutaneous flushing. It has been shown that pyrazoles act as partial agonists for the nicotinic acid receptor. Richman et al. [105] postulated that tissue selectivity, commonly observed with partial agonists, could help prevent side effects on skin cells (i.e., reducing or eliminating flushing). The authors decided to test the hypothesis that there are GPR109A agonists that are potent anti-lipolytic agents that do not cause flushing. It was shown that biaryl cyclohexene carboxylic acids act as full and potent niacin receptor (GPR109A) agonists. Compound **41** (Table 5), as β^2,3^-amino acid derivative, displayed excellent receptor activity, good PK across species ((EC_50_ = 4.6 μM in the GTPγS assay for the rat NA receptor and 1.3 μM in the GTPγS assay for the dog NA receptor), remarkably clean off-target profiles, good ancillary pharmacology and was highly potent in vivo in comparison to niacin [106].

## 3. Conclusions

This manuscript provides an overview of the current state of knowledge about selected β, β^2^, β^3^ and β^2,3^-amino acid moiety-containing heterocycles. In recent years, they have been successfully applied as antiviral, anti-inflammatory and antibacterial compounds, ALK inhibitors, agonists and antagonists of the receptors and their state-of-the-art applications in these fields have expanded enormously, proving their utility. Until now, more than 200 different heterocycles containing a β-amino acid moiety have been synthesized and characterized in regard to their biological activity. Research on these compounds has significantly increased the knowledge of their synthesis and activity alike. Currently, most of them are promising agents for medicinal chemistry and new drugs. Due to this, there is still a strong need for the development of new methods of their synthesis. They may serve as a scaffolding for designing and synthesizing new biologically active compounds, drug precursors and even drugs themselves. It may also be speculated that most of the presented β-amino acid moiety-containing heterocycles can be applied as new, nonproteinogenic amino acid residues in peptide synthesis. This may make it possible to prepare a new class of biologically active compounds and new types of enzyme substrates or inhibitors. Therefore, this field of heterocyclic chemistry is still under extensive investigation.

## Figures and Tables

**Table 1:** Schematic presentation of β^2,3^-amino acid heterocyclic derivatives with antiviral activity.

**Compound 1** **A-87380**	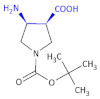	Neuraminidase inhibitor
**Compound 2** **A-192558**	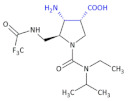	Neuraminidase inhibitor
**Compound 3**	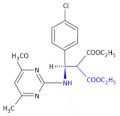	Antiviral activity against tobacco mosaic virus
**Compound 4**	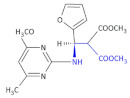	Antiviral activity against tobacco mosaic virus
**Compound 5**	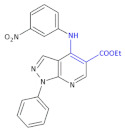	Anti-HSV-1 activity
**Compound 6** **a**: 1-phenyl-4-(phenylamino)-1*H*-pyrazolo-[3,4-*b*]-pyridine-5-carboxylic acid **b**: 4-(3-chlorophenylamino)-1-phenyl-1*H*-pyrazolo-[3,4-*b*]-pyridine- 5-carboxylic acid **c**: 4-(3-methoxyphenylamino)-1-phenyl-1*H*-pyrazolo-[3,4-*b*]-pyridine-5-carboxylic acid	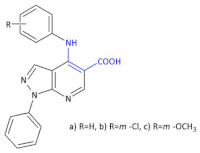	Antiviral activities against the VSV and Mayaro virus
**Compound 7**	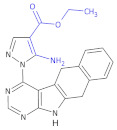	Anti-HAV activity
**Compound 8**	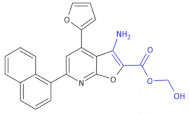	Antiviral activities toward the rotavirus Wa and adenovirus type 7 strains
**Compound 9**	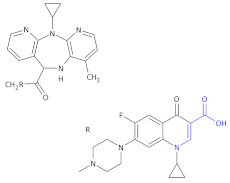	Anti-HIV activity, inhibitor of *Mycobacterium tuberculosis*
**Compound 10**	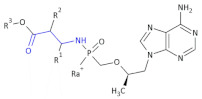	Inhibitor of ΗΓν reverse transcriptase

**Table 2:** 4-chlorobenzoylpyrrole derivatives acting as HIV-1 inhibitors [32].

Compound 11	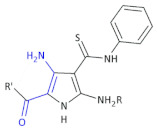		HIV-1 Inhibitors
	R	R’	RNase H ^a^IC_50_ (μM)
**A1**	4-CH_3_C_6_H_4_	C_6_H_5_	6.1 ± 1.6
**A2**	4-CH_3_C_6_H_4_	4-CH_3_C_6_H_4_	8.2 ± 1.7
**A3**	4-CH_3_C_6_H_4_	4-OCH_3_C_6_H_4_	8.0 ± 2.6
**A4**	4-CH_3_C_6_H_4_	4-ClC_6_H_4_	>100
**A5**	4-CH_3_C_6_H_4_	4-BrC_6_H_4_	8.3 ± 2.0
**A6**	4-CH_3_C_6_H_4_	3,4-Cl_2_C_6_H_3_	>100
**A7**	4-OCH_3_C_6_H_4_	CH_3_	>100
**A8**	4-OCH_3_C_6_H_4_	C_6_H_5_	19 ± 3
**A9**	4-OCH_3_C_6_H_4_	4-CH_3_C_6_H_4_	>100
**A10**	4-OCH_3_C_6_H_4_	4-OCH_3_C_6_H_4_	>100
**A11**	4-OCH_3_C_6_H_4_	4-BrC_6_H_4_	>100
**A12**	4-OCH_3_C_6_H_4_	4-ClC_6_H_4_	>100
**A13**	4-OCH_3_C_6_H_4_	3,4-Cl_2_C_6_H_3_	>100
**A14**	4-ClC_6_H_4_	C_6_H_5_	20 ± 4
**A15**	4-ClC_6_H_4_	4-CH_3_C_6_H_4_	9.0 ± 2.0
**A16**	4-ClC_6_H_4_	4-OCH_3_C_6_H_4_	8.0 ± 2.1
**A17**	4-ClC_6_H_4_	4-ClC_6_H_4_	8.2 ± 2.4
**A18**	*i*-C_3_H_7_	C_6_H_5_	>100
**A19**	C_6_H_5_CH_2_	C_6_H_5_	18 ± 3
**A20**	4-OCH_3_–2-pyridyl	C_6_H_5_	>100

**Table 3:** Schematic presentation of β^3^ and β^2,3^-amino acid heterocyclic derivatives with anti-inflammatory activity.

**Compound 12** **Zomerpirac**	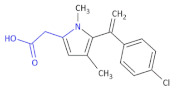	Prostaglandin synthetase inhibitor
**Compound 13** **Tolmetin**	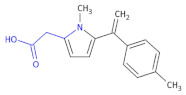	Prostaglandin synthetase inhibitor
**Compound 14** **Ketorolac**	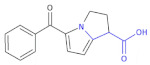	Prostaglandin synthetase inhibitor
**Compound 15**	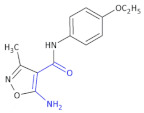	Anti-inflammatory and antibacterial activity
**Compound 16**	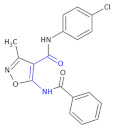	Anti-inflammatory and antibacterial activity
**Compound 17** **MZO-2**	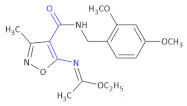	Anti-inflammatory activity, an inhibitor of the expression of caspases 3, 8, and 9 in Jurkat cells

**Table 4:** Schematic presentation of β-, β^2^-, β^3^- and β^2,3^-amino acid heterocyclic derivatives with antibacterial activity.

**Compound 18** **Moxifloxacin**	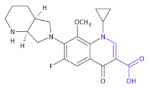	Activity against *Chlamydia pneumonia*
**Compound 19** **Ciprofloxacin**	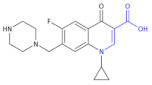	Activity against *Chlamydia trachomatis*
**Compound 20** **Oxetin**	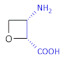	Inhibits *Bacillus subtilis* and *Pyricularia oryzae* exhibits a herbicidal activity and inhibits glutamine synthetase from spinach leaves
**Compound 21**	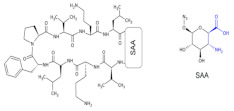	Not active activity against bacterial strains
**Compound 22**	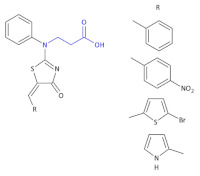	Activity against the strains of *S.* *aureus*, *B. cereus*, *E. coli* and *P.* *aeruginosa*
**Compound 23**	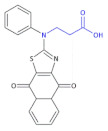	Activity against the strains of *S.* *aureus*, *B. cereus*, *E. coli* and *P.* *aeruginosa*
**Compound 24 a**: 3-(6,11-dioxo-6*H*-benzo[*b*] phenoxazin- 12(11*H*)-yl)butanoic acid **b**: 3-(2-methyl-6,11-dioxo-6*H* -benzo[*b*] phenoxazin-12(11*H*)-yl)butanoic acid **c**: 3-(2-chloro-6,11-dioxo -6*H*-benzo [*b*] phenoxazin-12(11*H*)-yl)butanoic acid	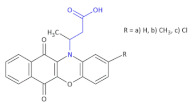	Activity against the *S.* *aureus* and *M. luteum* Gram-positive bacteria, inhibition of growth of *A. Niger* microorganisms, activity against *E. coli* (Compound **25**c)
**Compound 25**	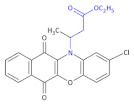	Activity against the *S.* *aureus* and *M. luteum* Gram-positive bacteria
**Compound 26**	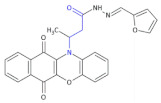	Activity against the *S.* *aureus* and *M. luteum* Gram-positive bacteria, inhibition of growth of *A. Niger* microorganisms
**Compound 27**	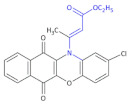	Activity against *M. Luteum*, *E. coli,* inhibition of growth of *A. Niger* microorganisms
**Compound 28**	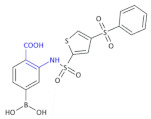	AmpC β-lactamase inhibitor
**Compound 29**	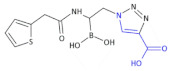	Inhibits *K. pneumoniae* β-lactamases KPC-2 and SHV-1
**Compound 30**	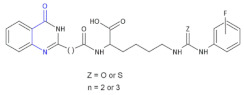	Antimicrobial activity
**Compound 31**	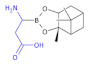	Serine protease inhibitor

**Table 5:** Schematic presentation of selected β^2^ and β^2,3^-amino acid heterocyclic derivatives characterized as agonists and antagonists of receptors.

**Compound 32**	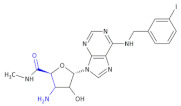	Agonist of the human adenosine A_3_ receptor
**Compound 33**	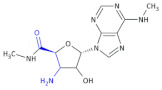	Agonist of the human adenosine A_3_ receptor
**Compound 34** **CP608039**	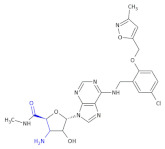	Selective adenosine A_3_ receptor agonist with 1,260-fold selectivity for the human A_3_ versus human A_1_ receptor
**Compound 35**	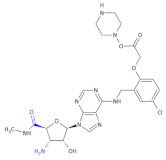	Full agonist of the A_3_ receptor
**Compound 36**	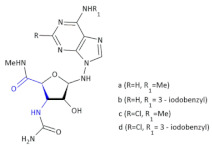	Agonists of the human adenosine A_3_ receptor
**Compound 37**	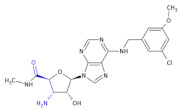	Agonist of the human adenosine A_3_ receptor
**Compound 38**	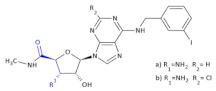	Agonist of the human adenosine A_3_ receptor
**Compound 39**	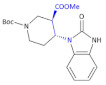	Nociceptin antagonist
**Compound 40**	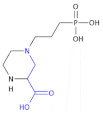	Selective NMDA glutamate receptor antagonist
**Compound 41**	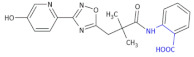	Niacin receptor agonist
